# Supplementary material for: TRPV4 and KRAS and FGFR1 gain-of-function mutations drive giant cell lesions of the jaw
Source: Nat Commun. 2018 Nov 1;9:4572. doi: 10.1038/s41467-018-06690-4 (PMC6212533; doi:10.1038/s41467-018-06690-4)
Supplement: Supplementary file 1 — Supplementary Information [file 41467_2018_6690_MOESM1_ESM.pdf]

TRPV4 and *KRAS* and *FGFR1* gain-of-function mutations drive giant cell lesions of the jaw

Supplementary Information

Gomes *et al.*

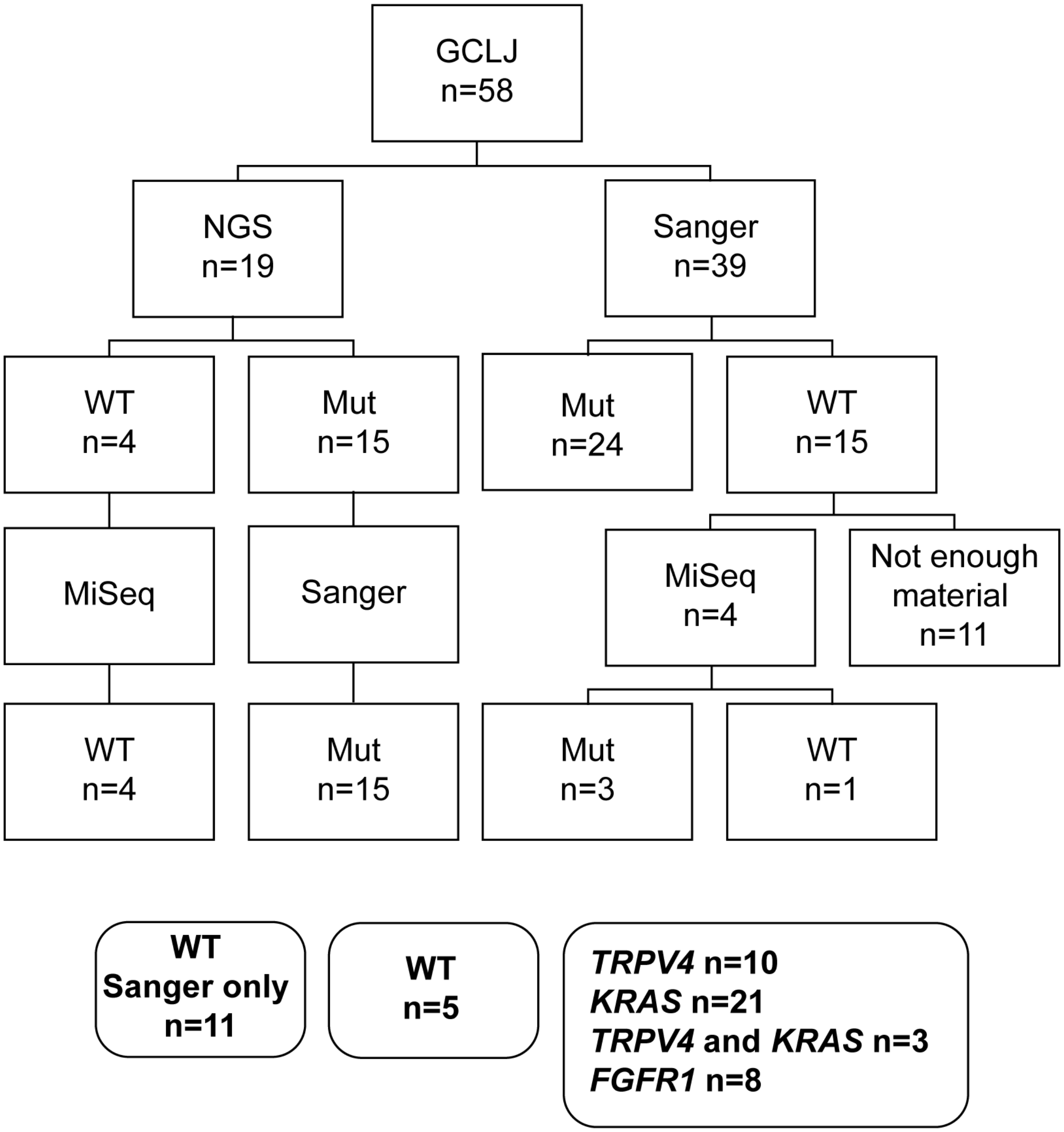

Supplementary Fig. 1. Flowchart summarizing the sequencing approach in this study.
Fifty-eight giant cell lesion of the jaw (GCLJ) samples were submitted to Next Generation Sequencing (NGS) or Sanger sequencing. Subsequently, WT samples with sufficient material (8/19) were deep sequenced by MiSeq to confirm WT status. A total of 42 GCLJ cases were mutant for *TRPV4*, *KRAS*, or *FGFR1*, whereas 5 samples were confirmed to be WT.

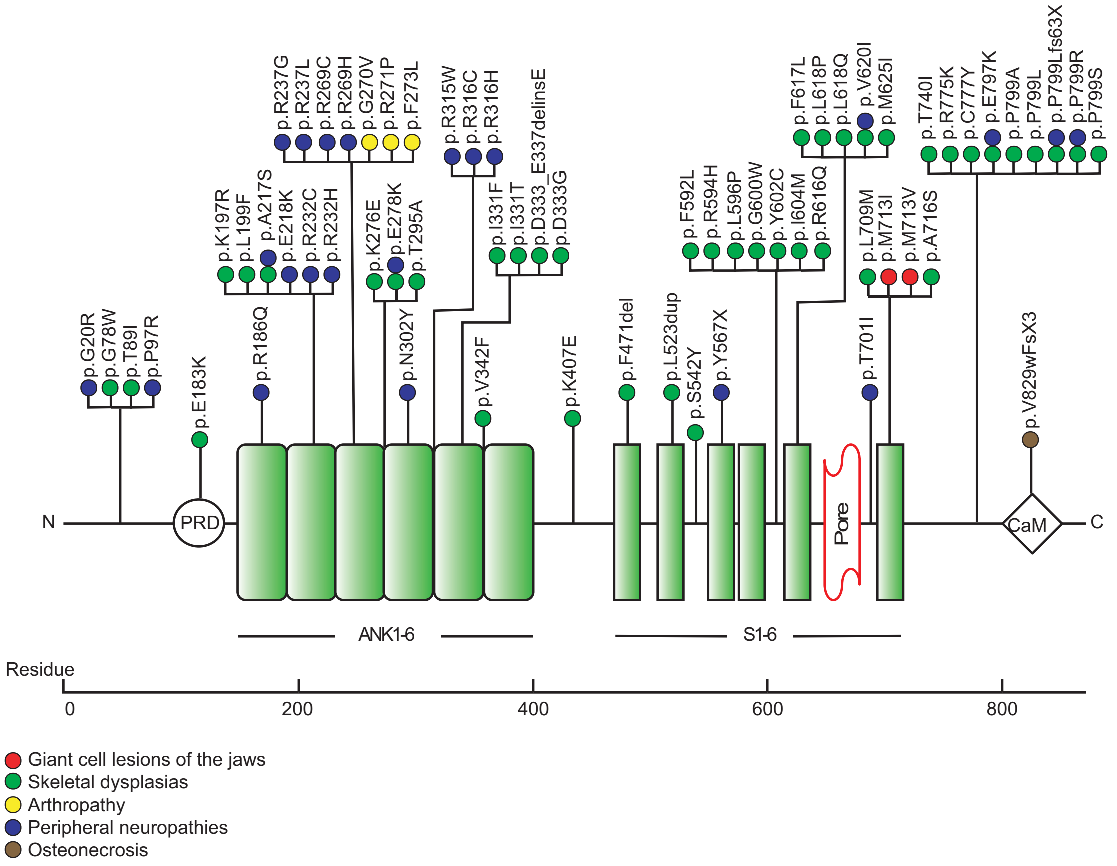

Supplementary Fig. 2. Schematic diagram of the TRPV4 channel and mutations associated with disease. The schema is composed of transmembrane segments (S1-6), pore-forming region, Ankyrin repeat domains (ANK1–6), proline rich domain (PRD) and calmodulin (CaM)-binding site. Each colored circle represents a disease or a phenotypic spectrum of a disease associated with *TRPV4* mutations. Red circle indicates the giant cell lesions of the jaw-associated mutations identified in this study. Green, yellow, blue and brown circles indicate, respectively, mutations previously reported in skeletal dysplasias (brachyolmia type 3, spondylometaphyseal dysplasia Kozlowski type, spondyloepimetaphyseal dysplasia Maroteaux pseudo-Morquio Type 2, parastremmatic dysplasia, metatropic dysplasia), arthropathy (familiar digital artropathy brachydactyly), peripheral neuropathies (Charcot-Marie-Tooth disease type 2C, congenital distal spinal muscle atrophy, scapuloperoneal spinal muscle atrophy) and osteonecrosis. Adapted from Nilius & Voets (2013).[1]

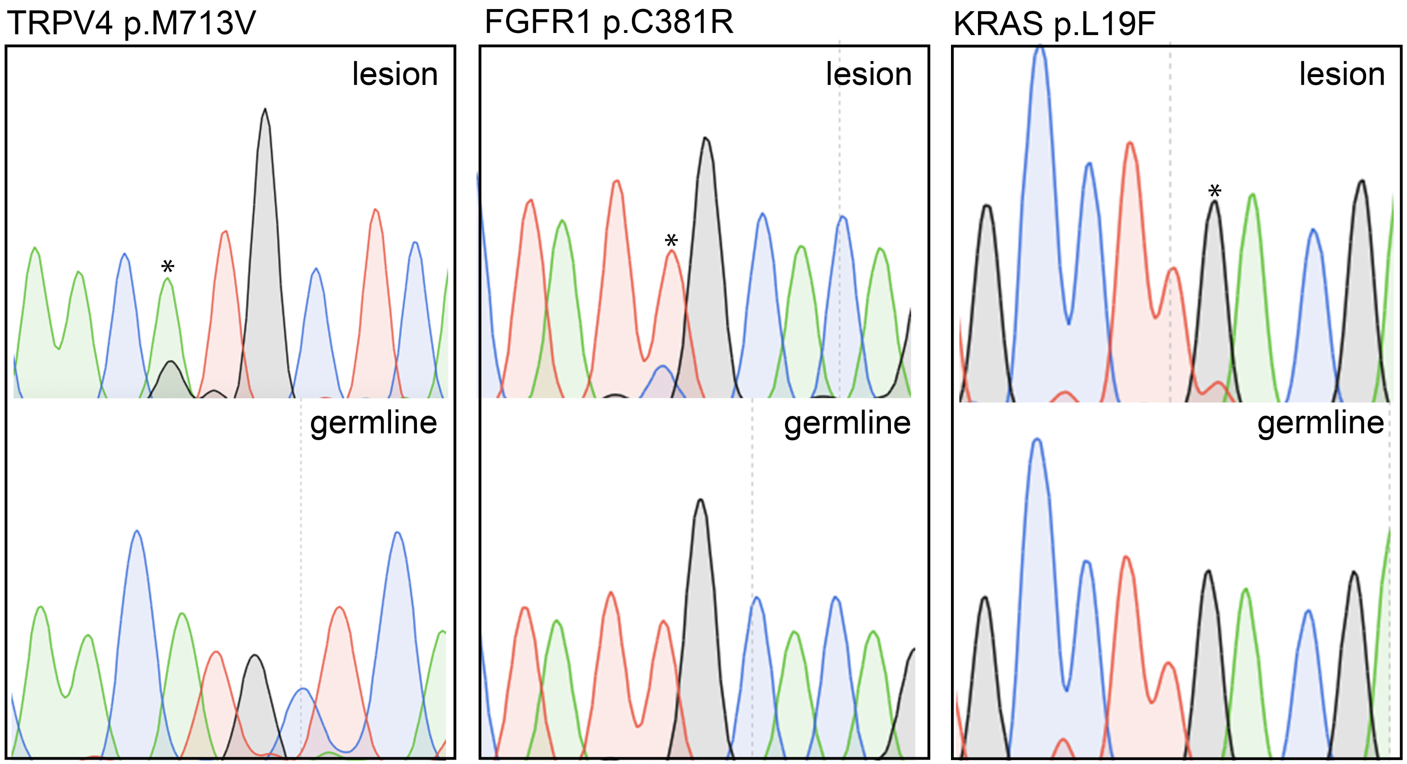

Supplementary Fig. 3. Somatic mutations in *TRPV4*, *FGFR1* and *KRAS*. Sanger sequencing chromatograms showing *TRPV4* p.M713V, *FGFR*1 p.C381R and *KRAS* p.L19F mutations in GCLJ (samples #1, #36 and #15, respectively). Mutations are marked by asterisks. The chromatograms for the matched germline DNA are also shown. The *TRPV4* and *FGFR1* mutations were confirmed by RNA-Sequencing and the *KRAS* mutation was confirmed by next generation sequencing (MiSeq).

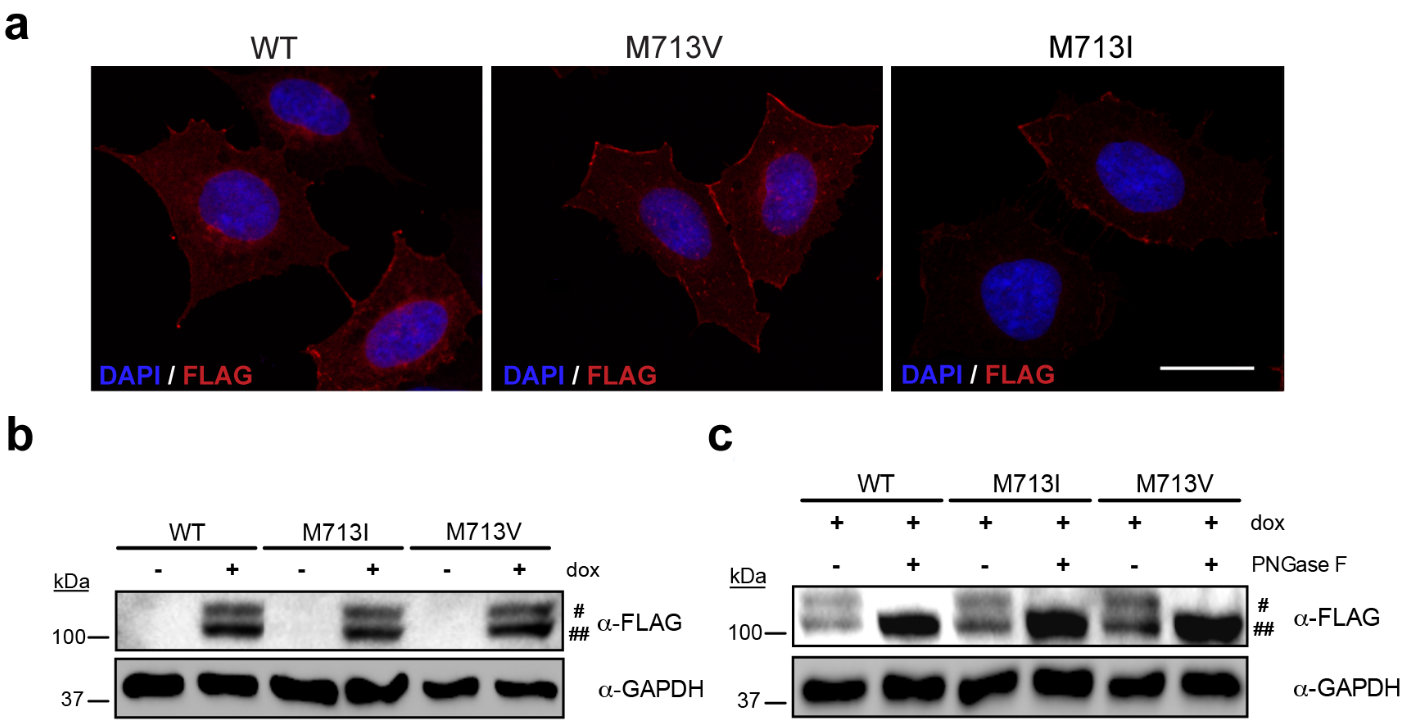

Supplementary Fig. 4. Expression of exogenous wild-type (WT) and mutant TRPV4 in HEK293 cells. a Confocal immunofluorescence and **b** immunodetection of FLAG-tagged WT-, M713V- and M713I-TRPV4 in HEK293 cells. **c** The lysates from TRPV4-expressing cells in **b** were submitted to N-deglycosylation assay using the PNGase F enzyme; glycosylated TRPV4, #; non-glycosylated TRPV4, ##. TRPV4 expression in HEK293 cells was induced with doxycyclin (dox) 24h prior to fixation or lysing of the cells. Scale bar, 20 μm.

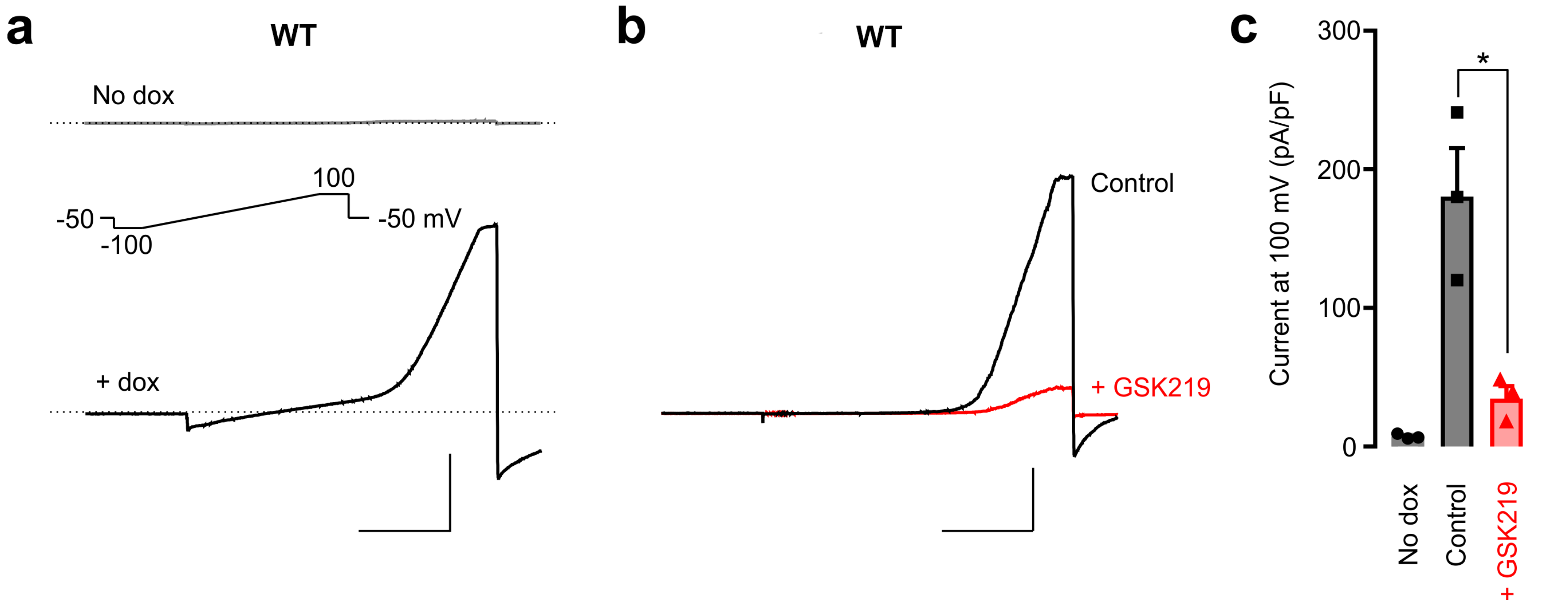

Supplementary Fig. 5. Characterization of current in HEK293 cells expressing exogenous wild-type TRPV4. a Representative traces of wild-type (WT) TRPV4 currents recorded in HEK293 cells using the conventional whole-cell configuration and 300-ms voltage ramps (from -100 to 100 mV, *inset*). Traces were recorded in two cells, the upper trace from a HEK293 cell without doxycycline (dox), and the lower is representative of cells where TRPV4 expression was induced with dox 24 h prior to recording. **b** Trace currents recorded from a WT TRPV4-HEK293 cell using the same protocol as in **a** while ruthenium red (RuR, 1 µM) was added to the bath solution. Recordings were made before (control, *black*) and after (*red*) the application of the TRPV4 channel blocker GSK2193874 (GSK219, 1 µM). Vertical scale bar, 50 pA/pF; horizontal scale bar, 100 ms. **c** Averaged summary data of outward current recorded at 100 mV in WT TRPV4 HEK293 cells cultured without dox (black circles), with dox (black squares), or with dox and after the application of GSK219 (red triangles). Data are presented as means ± s.e.m. (**P* < 0.05, paired Student's t-test, n = 3 each).

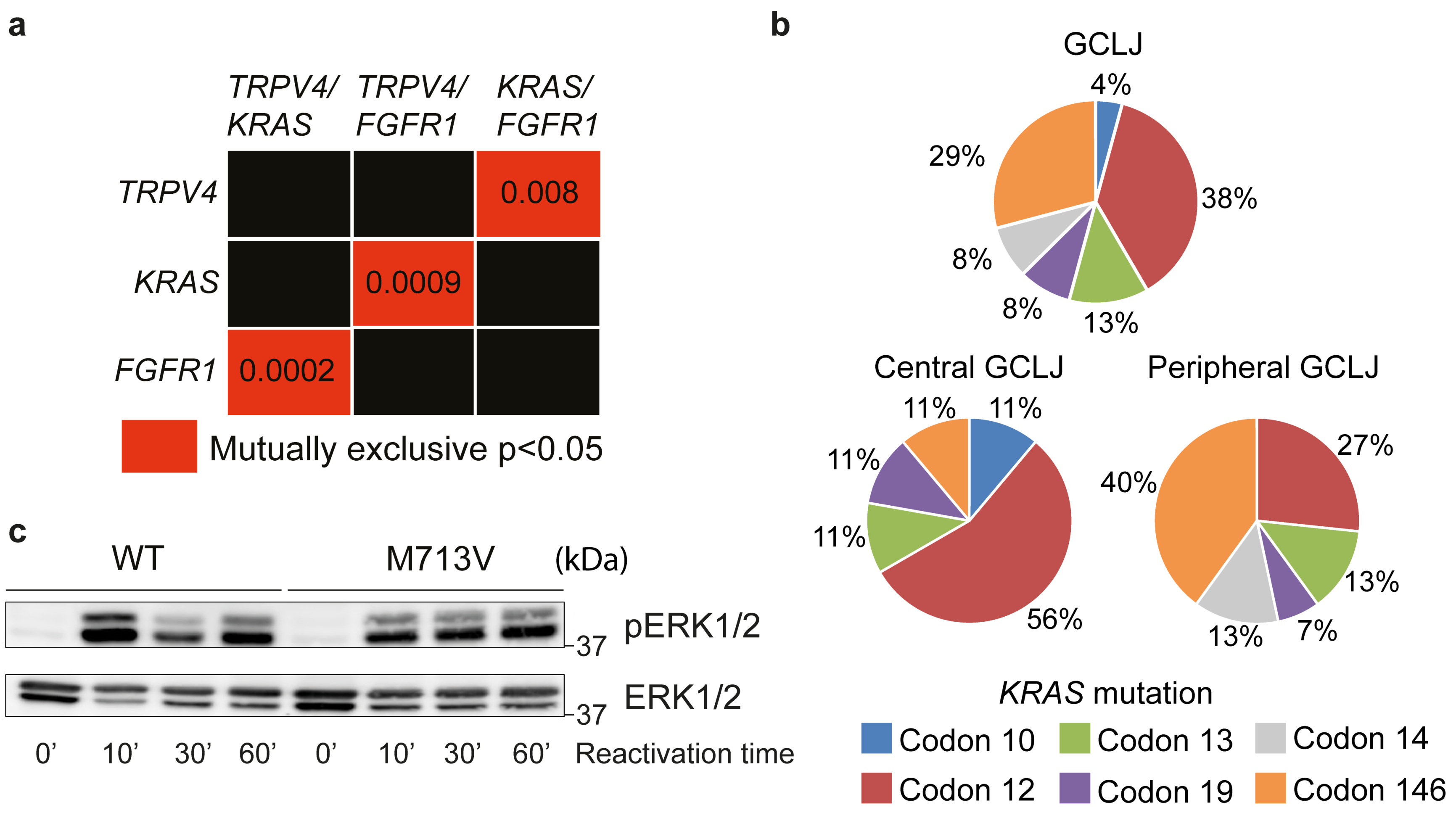

Supplementary Fig. 6. a Mutual exclusivity of *TRPV4*, *KRAS* and *FGFR1* mutations. P-values calculated utilizing Fisher´s Exact Test (two-sided) with values indicating statistically significant ($p<0.05$) preferences for mutual exclusivity. **b** The spectrum of *KRAS* mutations in GCLJ is shown on top, with codons 12 (9/24) and 146 (7/24) being the most frequently affected ones. While most *KRAS* mutations in the central variant of GCLJ (5/9) affected codon 12, the most frequent *KRAS* mutations in the peripheral variant occurred in codon 146 (6/15). **c** Immunoblot showing sustained phospho-ERK1/2 (pERK1/2) activation in *TRPV4* M713V HEK293 mutant cells compared to WT. A representative experiment of three independent assays is provided.

Fig. 3e

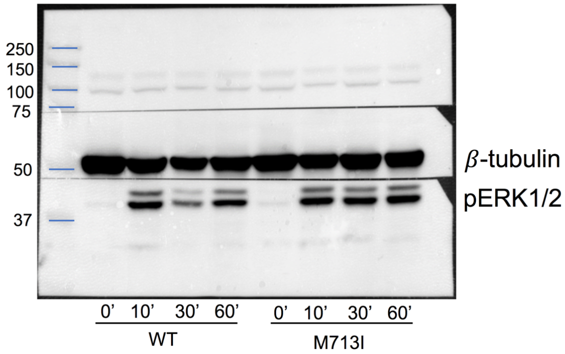

Supplementary Fig. 6c

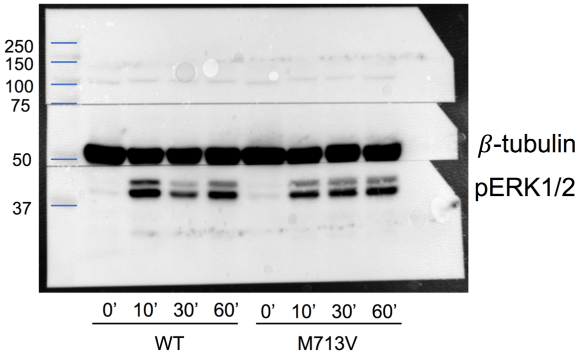

Fig. 3e

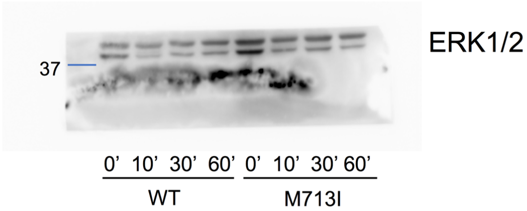

Supplementary Fig. 6c

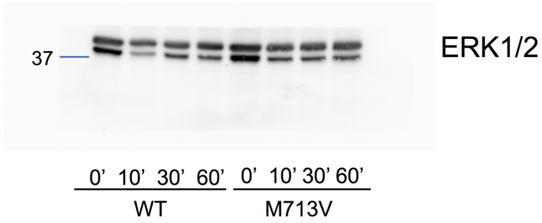

Supplementary Fig. 4b

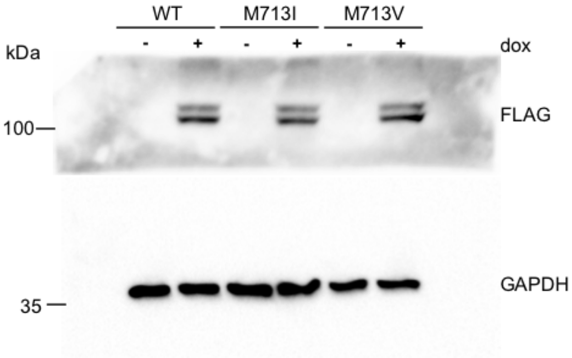

Supplementary Fig. 4c

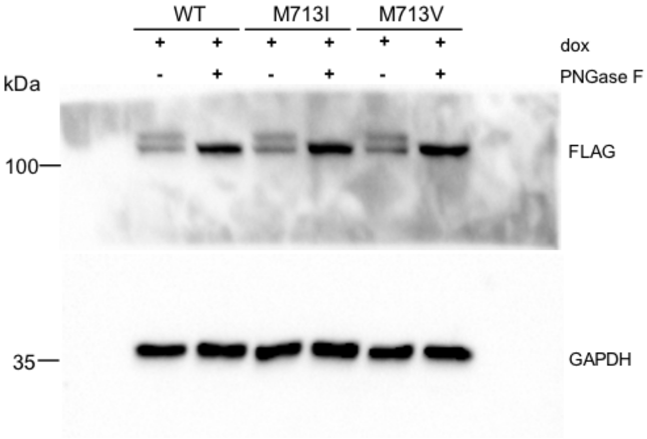

Supplementary Fig. 7. Uncropped scans of the Western blots in this study.

Supplementary References

1. Nilius, B. & Voets, T. The puzzle of TRPV4 channelopathies. *EMBO reports* **14**, 152-163 (2013).
