# Supplementary material for: TRPV4 and KRAS and FGFR1 gain-of-function mutations drive giant cell lesions of the jaw
Source: Nat Commun. 2018 Nov 1;9:4572. doi: 10.1038/s41467-018-06690-4 (PMC6212533; doi:10.1038/s41467-018-06690-4)
Supplement: Supplementary file 3 — Description of Additional Supplementary Files [file 41467_2018_6690_MOESM3_ESM.docx]

**Description of Additional Supplementary Files**

File Name: Supplementary Data 1

Description: A summary of clinical characteristics, Next Generation Sequencing (WES/RNA-Seq), Sanger Sequencing, MiSeq and immunohistochemistry (IHC) of giant cell lesions of the jaw samples.

File Name: Supplementary Data 2

Description: Tumor mutation burden (TMB) for a subgroup of giant cell lesions of the jaw analyzed in this study.

File Name: Supplementary Data 3

Description: List of variants detected by RNA-Seq in giant cell lesions of the jaw samples analyzed in this study.

File Name: Supplementary Data 4

Description: Whole Exome Sequencing coverage in 18 giant cell lesion of the jaw and 5 matched normal DNA samples from this study.

File Name: Supplementary Data 5

Description: Variants detected by Whole Exome Sequencing in 18 giant cell lesions of the jaw and 5 matched normal DNA samples from this study.

File Name: Supplementary Data 6

Description: List of somatic missense and frameshift TRPV4 mutations reported in cancer and extracted from COSMIC (Catalogue of Somatic Mutations in Cancer).
